# Dual antiplatelet therapy before coronary artery bypass grafting in patients with myocardial infarction: a prospective cohort study

**DOI:** 10.1186/s12893-021-01436-4

**Published:** 2021-12-31

**Authors:** Roxana Sadeghi, Mohammad Haji Aghajani, Reza Miri, Naser Kachoueian, Amir Nasser Jadbabaei, Mohammad Parsa Mahjoob, Fatemeh Omidi, Mahboobeh Ghazanfarabadi, Arash Sarveazad

**Affiliations:** 1 Prevention of Cardiovascular Disease Research Center, Shahid Beheshti University of Medical Sciences, Tehran, Iran; 2 Department of Cardiac Surgery, Shahid Beheshti University of Medical Sciences, Tehran, Iran; 3 Colorectal Research Center, Iran University of Medical Sciences, Tehran, Iran; 4 Nursing Care Research Center, Iran University of Medical Sciences, Tehran, Iran

**Keywords:** Dual anti-platelet therapy, Coronary artery bypass, Acute coronary syndrome, Aspirin, Clopidogrel

## Abstract

**Background:**

Dual antiplatelet therapy (DAPT) in patients with MI who are candidates for early coronary artery bypass grafting (CABG) can affect intraoperative and postoperative outcomes. Therefore, the aim of this study was to evaluate the effect of DAPT up to the day before CABG on the outcomes during and after surgery in patients with MI.

**Methods:**

In this prospective cohort study, 224 CABG candidate patients with and without MI were divided into two groups: (A) patients without MI who were treated with aspirin 80 mg/day before surgery (noMI-aspirin group; n = 124) and (B) patients with MI who were treated with aspirin 80 mg/day before surgery and clopidogrel (Plavix brand) at a dose of 75 mg/day (MI-DAPT group; n = 120). Dual or mono-antiplatelet therapy continued until the day before surgery. Patients were followed to assess in-hospital and 6-months outcomes.

**Results:**

The in-hospital mortality in MI-DAPT group was similar with noMI-aspirin group (OR 4.2; 95% CI 0.9–20.5; p = 0.071). The prevalence of CVA (p = 0.098), duration of hospital stay (p = 0.109), postoperative ejection fraction level (p = 0.693), diastolic dysfunction grade (p = 0.651) and postoperative PAP level (p = 0.0364) did not show difference between two groups. No mild or severe bleeding was observed in the patients. Six-month follow up showed that number of readmissions (p = 0.801), number of cases requiring angiography (p = 0.100), cases requiring re-PCI (p = 0.156), need for re-CABG (p > 0.999) and CVA (p > 0.999) did not differ between the two groups. During the 6-month follow-up, out-hospital mortality did not differ significantly between the two groups (p = 0.446).

**Conclusions:**

A 6-month follow-up showed that DAPT with aspirin and clopidogrel before CABG in patients with MI has no effect on postoperative outcomes more than mono-APT with aspirin. Therefore, DAPT is recommended in the preoperative period for these patients.

## Background

Coronary artery bypass grafting (CABG) was developed in 1962 as a method to bypass the blocked part of the coronary artery and improve blood flow to the heart. CABG is still used as a treatment in some of patients with acute coronary syndrome (ACS) [1]. ACS refers to a set of signs and symptoms including: unstable angina, ST (STEMI) and none-ST-segment elevation myocardial infarction (NSTEMI). Primary management for ACS involves optimal coronary artery blood flow through percutaneous coronary intervention (PCI) [2, 3]. CABG is an alternative option in special cases and in patients who have initially undergone PCI and re-referred for ACS management. In general, three groups of patients are the target population for CABG: (A) Patients with ACS and ischemia in which the artery responsible for myocardial ischemia is not suitable for PCI, (B) patients with ACS who have previously undergone PCI and return to the emergency, or patients who have an ischemic lesion in myocardium due to conditions such as severetriple-vessel disease or left main stem stenosis and (C) patients with ACS in whom PCI was incomplete, inadequate, or even unsuccessful [4].

If the hemorrhagic side effects of CABG are severe and require a blood transfusion, it certainly increases mortality, which contrasts with the long-term benefits of CABG. Bleeding after CABG is strongly influenced by the drugs prescribed before and after this surgery. Anticoagulants are among the most important drugs in this field [5, 6]. Therefore, adopting an appropriate strategy for the use of antiplatelet therapies in CABG candidate patients is very important. The basis of ACS treatment is the use of dual antiplatelet therapy (DAPT) using a nonsteroidal anti-inflammatory drug (aspirin) combined with an adenosine diphosphate receptor antagonist (such as clopidogrel) to reduce thrombotic and ischemic events [7–9]. Despite the benefits of DAPT in the management of CABG candidate patients, this strategy also has its risks, as it can increase the risk of bleeding up to 50%. If one antiplatelet drug (Aspirin or clopidogrel) is used, the risk is 20% [10, 11]. The amount of blood lost after CABG is a very important factor in the survival rate of MI patients [12–15]. Therefore, in the optimal management of MI patients who are candidates for CABG, it is necessary to answer the question of how much DAPT before CABG (compared to one antiplatelet) can affect the postoperative outcomes. The present prospective cohort study was designed to evaluate the effect of DAPT with aspirin and clopidogrel up to the day before CABG surgery on postoperative outcomes in patients with ACS.

In this study, primary objectives include assessment of postoperative bleeding, intraoperative and postoperative blood and pack cell transfusions, cardio-bypass and cross-clamp time and surgical re-exploration. Secondary objectives include assessment of mortality, cerebrovascular accident (CVA), length of hospitalization and stay in cardiac surgery intensive care unit (CSICU), ejection fraction (EF) rate, hospital costs and need for readmission, re-angiography, re-PCI and CABG at 6-month follow-up, recurrence and occurrence of stroke or death.

## Methods

### Study design and patient selection

The present single-center, nonrandomized, unblinded prospective cohort study included 244 candidates for CABG surgery between 21/03/2018 and the 21/03/2020. All methods were performed in accordance with the Declaration of Helsinki regulations. The definition of MI was based on the universal definition of myocardial infarction in 2018 [16]. The surgical and treatment process was performed in Imam Hossein Educational and Medical Center, Shahid Beheshti University of Medical Sciences, Tehran, Iran. The design and conduct of the study was approved by the Ethics Committee (Code: IR.SBMU.RETECH.REC.1399.547) of Shahid Beheshti University of Medical Sciences. Although the patient care process was performed in the hospital according to the current standard protocol from the moment of admission to the study, but before entering the study, written informed consent was obtained from all participants. All patients who underwent CABG during the 24-month study period and received aspirin or aspirin + clopidogrel until the day before surgery were included. Exclusion criteria were patients who required other cardiac surgery in addition to CABG, such as valve repair or replacement, patients taking preoperative anticoagulants or a history of hematological or mental disorders. Preoperative all demographic information (age, sex, height, weight, presence of classic risk factors for cardiovascular disease and preoperative medication), clinical features (blood pressure and heart rate at hospitalization, electrocardiogram, transthoracic echocardiographic findings including EF, diastolic function, valvular problems and pulmonary artery pressure), biochemical data (Hemoglobin, Platelets, White blood cell, Creatinine, Lipid profile, Fasting Blood Sugar, HbA1C) and angiographic characteristics were recorded.

### Grouping and antiplatelet therapy

Patients were divided into two groups:

(A) Aspirin group (n = 124): Who were hospitalized without MI and treated with aspirin 80 mg/day before surgery.
(B) DAPT group (n = 120): Who were hospitalized with MI and received Aspirin + Clopidogrel (aspirin 80 mg/day before surgery and Plavix brand of clopidogrel at a dose of 75 mg/day) until the day before surgery and received 600 mg of clopidogrel as a loading dose at the time of admission.

Both groups received post-operative DAPT according to treatment standards, and it was recommended that DAPT be continued for up to 1 year after surgery [17–22].

### CABG procedure

Using routine protocol, anesthesia and CABG surgery were performed in all patients briefly as follows. All surgeries were performed by two surgeons. The patient received morphine (0.05 mg/kg) one hour before surgery. Prior to anesthesia, a CV catheter in the internal jugular vein and a radial artery cannula was implanted to monitor the patient. Anesthesia was induced. Body temperature was maintained between 32 and 37 °C during surgery. After median sternotomy, before the onset of cardiopulmonary bypass (CPB) and during anastomosis, activated clotting time (ACT) > 450 s was established using a loading dose (250 U/kg) of heparin. Crystalloid cardioplegia was prescribed to protect the myocardium. Finally, all patients were transferred to the ICU without receiving antifibrinolytics. Platelet injection criteria was postoperative platelet count less than 50,000/mm^3^ with bleeding more than 200 mL/h for 2 consecutive hours. Need for pack cell transfusion was based on the clinical condition and hemoglobin value (in case of asymptomatic patients: Hemoglobin < 8 mg/dL, age < 65 years and mixed venous oxygen saturation (SVO2) > 65% and in case of symptomatic patients: Hemoglobin between 8 and 10 mg/dL, age > 65 years, EF < 50% and SVO2 < 65%). Surgical re-exploration was performed if chest tube drainage was more than 200 mL/h for 6 consecutive hours or more than 400 mL/h in the 1st hour (provided that the ACT and general coagulation status were normal).

### Outcomes

Primary outcomes in this study include postoperative bleeding (bleeding resulting in death, reoperation due to bleeding, intracranial haemorrhage, transfusion of 5 or more units of RBCs over 48 h, and chest tube drainage in excess of 2000 mL over 24 h was considered severe bleeding and less chest tube drainage was considered mild), intraoperative and postoperative blood transfusions, cardio-bypass time, cross-clamp time, number of pack cell transfusion, and surgical re-exploration. Secondary outcomes include mortality, CVA, length of hospital stay in CSICU and total length of hospital stay, EF rate, hospital costs and need for readmission, need for re-angiography, re-PCI, need for CABG at 6-month follow-up, recurrence and occurrence of stroke or death.

### Data analysis

Data were analyzed in STATA 14.0 statistical program. Quantitative variables were reported as mean and standard deviation and qualitative variables were reported as frequency and percentage. Comparison of quantitative variables between Aspirin alone and Aspirin + clopidogrel groups was performed using t-test or Kuroscal Wallis test and qualitative variables were performed using Chi-square test or Fisher’s exact test. Since EF before surgery had a significant difference between the two groups, the status of EF in the follow-up period was compared between the two groups using ANCOVA test with adjustment for preoperative EF values. To investigate the relationship between preoperative Aspirin + clopidogrel administration and in-hospital mortality of the patients, a multivariate logistic regression or multivariate linear logistic regression was performed for confounders. In these two models, the findings were reported as odds ratio (OR) or beta coefficient with 95% confidence interval (95% CI), respectively. In all analyzes, p value less than 0.05 was considered as a significant level.

## Results

### Demographic and baseline characteristics

Data from 244 patients were included in the present study. The mean age of these patients was 60.4 ± 9.2 years and 70.5% of the patients were men. 124 patients in the aspirin group and 120 patients in the DAPT group were included. There was no difference in age (p = 0.097) and sex (p = 0.868) distribution of patients in the two groups. In the study of baseline variables of patients before surgery, it was found that education level (p = 0.009), body mass index (p = 0.001), prevalence of dyslipidemia (p = 0.020), prevalence of COPD (p = 0.012), history of PCI (p = 0.031), history of aspirin use before hospitalization (p = 0.001), history of statins (p < 0.001) were significantly different between the two groups. The level of education in the aspirin group was higher than in the DAPT group (Table 1). The prevalence of overweight and obesity was higher in the DAPT group (65.7%) than in the aspirin group (53.2%). A history of dyslipidemia (18.6% vs. 8.3%) was more in the aspirin group, while the prevalence of COPD (16.8% vs. 6.4%) and previous PCI (18.3% vs. 8.8%) was higher in patients in the DAPT group. The prevalence of aspirin (62.9% vs. 42.5%) and statins (53.2% vs. 29.2%) consumption in the past was higher in the aspirin group. Laboratory findings and preoperative imaging of patients showed that the hemoglobin A1c level in the aspirin group was significantly higher than the DAPT group (8.6 ± 2.2 vs. 7.4 ± 2.1 mg/dL). The mean EF level in the aspirin group was slightly higher than the DAPT group (46.9% vs. 44.0%; p = 0.013). The prevalence of mitral regurgitation in the aspirin group was higher than the DAPT group (81.4% vs. 72.5%; p = 0.047). Electrocardiographic examination of patients showed that LBBB in the aspirin group was 9.7%, while in the DAPT group it was 3.3% (p = 0.045).

**Table 1:** Baseline and surgery related characteristics of the patients

Variable	Aspirin alone (n = 124)	DAPT (n = 120)	Total (n = 244)	p value
Age (year)	61.3 ± 9.0	59.4 ± 9.2	60.4 ± 9.2	0.097
Gender				
Female	36 (29.0)	36 (30.0)	72 (29.5)	0.868
Male	88 (71.0)	84 (70.0)	172 (70.5)	
Marital status				
Not married	9 (7.3)	7 (5.8)	16 (6.6)	0.653
Married	115 (92.7)	113 (94.2)	228 (93.4)	
Education				
Less than diploma	8 (6.7)	20 (17.5)	28 (12.0)	**0.009**^**a**^
Diploma	106 (88.3)	91 (79.8)	197 (84.2)	
Graduate	6 (5.0)	3 (2.6)	9 (3.8)	
Income				
< 3 million Tomans	5 (4.1)	11 (9.5)	16 (6.8)	0.089^a^
3–6 million Tomans	105 (86.8)	98 (84.5)	203 (85.6)	
> 6 million Tomans	11 (9.1)	7 (6.0)	18 (7.6)	
BMI (kg/m^2^)				
< 18.9	5 (4.0)	0 (0.0)	5 (2.0)	**0.001**^**b**^
18.9–24.9	53 (42.7)	40 (33.3)	93 (38.1)	
25–29.9	37 (29.8)	62 (51.7)	99 (40.6)	
> 30	29 (23.4)	18 (15.0)	47 (19.3)	
Physical activity (times/week)				
0–1 time	42 (34.2)	31 (25.8)	73 (30.0)	0.062
2–4 times	61 (49.6)	55 (45.8)	116 (47.7)	
≥ 5 times	20 (16.3)	34 (28.3)	54 (22.2)	
Substance abuse				
Smoking				
No smoker	59 (47.6)	47 (39.2)	106 (43.4)	0.257
Ex-smoker	18 (14.5)	15 (12.5)	33 (13.5)	
Current smoker	47 (37.9)	58 (48.3)	105 (43.0)	
Alcohol consumption	5 (4.0)	2 (1.7)	7 (2.9)	0.447^b^
Amphetamine	3 (2.4)	4 (3.33)	7 (2.9)	0.719^b^
Family history of CAD	19 (15.3)	19 (15.8)	38 (15.6)	0.912
Underlying disease^c^				
Dyslipidaemia	23 (18.6)	10 (8.3)	33 (13.5)	**0.020**
Hypertension	81 (65.3)	67 (55.8)	148 (60.7)	0.129
Diabetes	67 (54.0)	54 (45.0)	121 (49.6)	0.158
Renal failure	8 (6.4)	5 (4.2)	13 (5.3)	0.427
Dialysis	2 (1.6)	3 (2.5)	5 (2.0)	0.680^b^
PAD	5 (4.0)	4 (3.3)	9 (3.7)	> 0.999^b^
CVA	6 (4.8)	8 (6.7)	14 (5.7)	0.539
COPD	8 (6.4)	20 (16.8)	28 (11.5)	**0.012**
CAD	58 (46.8)	49 (40.8)	107 (43.8)	0.350
MI	17 (13.7)	19 (15.8)	36 (14.8)	0.640
Heart failure	4 (3.2)	3 (2.5)	5 (2.9)	> 0.999^b^
Previous PCI	11 (8.8)	22 (18.3)	33 (13.5)	**0.031**
Previous CABG	1 (0.8)	1 (0.8)	2 (0.8)	> 0.999^b^
Drug history^d^				
Aspirin	78 (62.9)	51 (42.5)	129 (52.9)	**0.001**
Clopidogrel	29 (23.4)	23 (19.2)	52 (21.3)	0.421
ACE/ARBi	64 (51.6)	50 (41.7)	114 (46.7)	0.120
Insulin	16 (12.9)	11 (9.2)	27 (11.1)	0.352
Statins	66 (53.2)	35 (29.2)	101 (41.4)	** < 0.001**
Beta-blockers	50 (40.3)	35 (29.2)	85 (34.8)	0.067
Ca-channel blocker	5 (4.0)	5 (4.2)	10 (4.1)	> 0.999^b^
Clinical findings in admission				
SBP (mmHg)	128.4 ± 20.5	126.3 ± 22.3	127.4 ± 21.4	0.467
DBP (mmHg)	76.2 ± 11.7	76.5 ± 11.9	76.3 ± 11.8	0.821
Heart failure	8 (6.45)	3 (2.5)	11 (4.5)	0.217^b^
Pulmonary edema	0 (0.0)	2 (1.7)	2 (0.8)	0.241^b^
Cardiogenic shock	0 (0.0)	1 (0.8)	1 (0.4)	0.492^b^
Syncope	2 (1.6)	1 (0.8)	3 (1.2)	> 0.999^b^
Laboratory findings before surgery				
Haemoglobin (mg/dL)	13.0 ± 1.7	13.0 ± 1.8	13.0 ± 1.8	0.764
Platelet (n/mm^3^)	222.0 ± 70.6	219.4 ± 65.2	220.8 ± 67.9	0.766
WBC (n/mm^3^)	9.3 ± 3.8	10.1 ± 3.1	9.7 ± 3.5	0.067
Cr (mg/dL)	1.2 ± 0.7	1.2 ± 0.8	1.2 ± 1.1	0.985
Total cholesterol (mg/dL)	152.4 ± 42.5	163.0 ± 42.3	158.0 ± 42.5	0.087
LDL (mg/dL)	94.8 ± 31.1	102.3 ± 31.8	98.8 ± 31.6	0.107
HDL (mg/dL)	34.7 ± 7.4	35.2 ± 9.8	34.9 ± 8.8	0.715
TG (mg/dL)	145.0 ± 99.9	141.0 ± 79.9	142.9 ± 89.6	0.763
FBS (mg/dL)	141.4 ± 53.4	131.7 ± 46.7	136.7 ± 50.4	0.143
HbA1c (%)	8.6 ± 2.2	7.4 ± 2.1	8.0 ± 2.2	**0.009**
ECG findings				
Complete heart block	0 (0.0)	2 (1.7)	2 (0.8)	0.241^b^
LBBB	12 (9.7)	4 (3.3)	16 (6.6)	**0.045**
RBBB	10 (8.1)	5 (4.2)	15 (6.2)	0.205
Echocardiography before surgery				
Ejection fraction (%)	46.9 ± 8.7	44.0 ± 9.5	45.4 ± 9.2	**0.013**
≥ 55%	35 (28.2)	24 (20.0)	59 (24.2)	**0.032**
40–54%	71 (57.3)	67 (55.8)	138 (56.6)	
35–40%	9 (7.3)	12 (10.0)	21 (8.6)	
< 35%	9 (7.3)	17 (14.2)	26 (10.7)	
Mitral regurgitation				
No	21 (16.9)	33 (27.5)	54 (22.1)	**0.047**^**b**^
Mild	101 (81.4)	87 (72.5)	188 (77.0)	
Moderate	2 (1.6)	0 (0.0)	2 (0.8)	
PAP	26.3 ± 7.8	25.7 ± 8.2	26.1 ± 8.0	0658
Angiographic properties				
Left main involvement				
No	110 (88.7)	100 (83.3)	210 (86.1)	0.225
Yes	14 (11.3)	20 (16.7)	34 (13.9)	
1VD	3 (2.4)	9 (7.5)	12 (4.9)	0.066^d^
2VD	16 (12.9)	20 (16.7)	36 (14.8)	
3VD	105 (84.7)	91 (75.8)	196 (90.3)	
Primary PCI	1 (0.8)	53 (44.2)	54 (22.1)	** < 0.001**
Using balloon pump	0 (0.0)	3(2.5)	3 (1.2)	0.117
CABG data				
Number of graft				
1	8 (6.4)	9 (7.5)	17 (7.0)	0.068^a^
2	17 (13.7)	26 (21.7)	43 (17.6)	
3	47 (37.9)	47 (39.2)	94 (38.5)	
4	46 (37.1)	33 (27.5)	79 (32.4)	
5	6 (4.9)	5 (4.2)	11 (4.5)	
LIMA usage	120 (96.8)	116 (96.7)	236 (96.7)	0.962
Off-pump surgery	10 (8.1)	16 (13.3)	26 (10.7)	0.182

Bold values are significant

Data are presented as n (%) or mean ± standard deviation

*ACE/ARB* angiotensin converting enzyme/angiotensin II receptor blockers; *BMI* body mass index; *CAD* coronary artery disease; *COPD* chronic obstructive pulmonary disease; *CVA* Cerebrovascular accident; *Cr* creatinine; *DBP* diastolic blood pressure; *ECG* electrocardiogram; *FBS* fasting blood sugar test; *HbA1c* hemoglobin A1C; *HDL* high-density lipoprotein; *LBBB* left bundle branch block; *LDL* low-density lipoprotein; *P-PCI* primary percutaneous coronary intervention; *RBBB* right bundle branch block; *TG* triglycerides; *WBC* white blood cells; *MI* myocardial infarction; *PAD* peripheral arterial disease; *PAP* pulmonary artery pressure; *PCI* percutaneous coronary intervention; *SBP* systolic blood pressure; *VD* vessel disease

^a^Based on Kruskal–Wallis test

^b^Based of Fisher’s exact test

^c^Since some patients had at least two underlying diseases, the total percentage is more than 100%

^d^Since some patients had at least two drug history, the total percentage is more than 100%

### In-hospital outcomes

Follow-up of patients after surgery showed that the level of hemoglobin before and after surgery at its lowest level was no different between the two groups of aspirin and DAPT (p = 0.502). No mild or severe bleeding was observed in the patients. The cases of blood transfusion during surgery in the aspirin group were slightly higher than the DAPT group, which did not show a difference between the two groups by multivariate logistic regression analysis (82.3% vs. 75.8%; OR 1.2; p = 0.681). Other variables of surgical time such as cardio-bypass time (p = 0.552) and cross-clamp time (0.979), number of pack cell transfusion (p = 0.560) were not different between the two groups. Follow-up of patients at the time of admission showed 11 deaths among patients, of which 2 (1.6%) were in the aspirin group and 9 (7.5%) were in the DAPT group. Although univariate analysis showed that the prevalence of mortality was higher in DAPT group than aspirin, but multivariate logistic regression test did not show any difference between the two groups (OR 4.2; 95% CI 0.9–20.5; p = 0.071). The prevalence of CVA after surgery did not show any difference between the two groups (p = 0.098). Although the duration of ICU hospitalization in the DAPT group was slightly longer than the aspirin group, no significant difference was observed between the groups in multivariate linear regression analysis (Beta = 0.7; 95% CI − 0.2 to 1.6; p = 0.109). Postoperative EF level (p = 0.693), diastolic dysfunction grade (p = 0.651) and postoperative PAP level (p = 0.0364) did not show any difference between the two groups. Hospital costs in the DAPT group were higher in univariate analyzes than in the aspirin group (p < 0.001), but multivariate linear regression showed that performing primary PCI is the main factor in increasing hospital costs in the DAPT group (Beta = 60.3; 95% CI 32.6–87.9; p < 0.001). DAPT administration had no effect on hospital costs (Beta = 22.7; 95% CI − 0.2 to 45.6; p = 0.052) (Table 2).

**Table 2:** In hospital outcome of patients treated with DAPT or aspirin alone

Parameter	Univariate			Multiple^d^		
	Aspirin alone	DAPT	p	OR/Coef.	95% CI	p
CABG in first day	2 (1.6)	8 (6.7)	0.057^b^	**–**	**–**	**–**
CABG after 24 h	122 (98.4)	112 (93.3)	0.057^b^	**–**	**–**	**–**
Cardio-bypass time (min)	108.7 ± 30.4	112.4 ± 56.3	0.552	**–**	**–**	**–**
Cross-clamp time (min)	66.7 ± 18.6	66.6 ± 29.3	0.979	**–**	**–**	**–**
Need to blood transfusion	110 (88.7)	101 (84.2)	0.300	**–**	**–**	**–**
Number of pack cell transfusion	2.4 ± 1.5	2.6 ± 1.8	0.560	**–**	**–**	**–**
Blood transfusion during surgery						
1 unit	88 (71.0)	88 (73.3)	**0.026**^**a**^	1.2	0.5, 3.0	0.681
2 units	12 (9.7)	3 (2.5)				
3 units	2 (1.6)	0 (0.0)				
Haemoglobin 1 day(mg/dL)	9.4 ± 1.8	9.5 ± 1.9	0.502	**–**	**–**	**–**
Haemoglobin 2 day(mg/dL)	10.3 ± 1.4	10.2 ± 1.3	0.930	**–**	**–**	**–**
Cr 1 day (mg/dL)	1.4 ± 1.1	1.4 ± 0.8	0.817	**–**	**–**	**–**
Cr 2 day (mg/dL)	1.3 ± 1.0	1.2 ± 0.9	0.889	**–**	**–**	**–**
Duration of IABP				**–**	**–**	**–**
No	124 (100.0)	116 (97.5)	0.117^b^	**–**	**–**	**–**
Yes	0 (0.0)	3 (2.5)				
Minor bleeding	0 (0.0)	0 (0.0)	**–**	**–**	**–**	**–**
Major bleeding	0 (0.0)	0 (0.0)	**–**	**–**	**–**	**–**
Retroperitoneal bleeding	0 (0.0)	0 (0.0)	**–**	**–**	**–**	**–**
Hematoma	0 (0.0)	0 (0.0)	**–**	**–**	**–**	**–**
GI bleeding	0 (0.0)	0 (0.0)	**–**	**–**	**–**	**–**
Repeated sternotomy	4 (3.2)	0 (0.0)	0.122^b^	**–**	**–**	**–**
Recurrent ischemia	0 (0.0)	0 (0.0)	**–**	**–**	**–**	**–**
Recurrent MI	0 (0.0)	1 (0.8)	0.496^b^	**–**	**–**	**–**
Stent thrombosis	0 (0.0)	1 (0.8)	0.492	**–**	**–**	**–**
CVA	2 (1.6)	7 (5.8)	0.098	**–**	**–**	**–**
Death	2 (1.6)	7 (5.8)	0.098	**–**	**–**	**–**
Death in cat-Lab	0 (0.0)	0 (0.0)	–	**–**	**–**	**–**
Later in-hospital death	2 (1.6)	9 (7.5)	**0.028**	4.2	0.9, 20.5	0.071
Hospital stay (day)	13.6 ± 5.4	15.6 ± 12.0	0.113	**–**	**–**	**–**
ICU stay (day)	5.0 ± 1.9	5.8 ± 3.4	**0.045**	0.7	− 0.2, 1.6	0.109
EF (%) after surgery	46.4 ± 8.9	44.4 ± 9.5	0.693^c^	**–**	**–**	**–**
≥ 55%	27 (23.9)	23 (21.1)	0.267^b^	**–**	**–**	**–**
40–54%	70 (62.0)	63 (57.8)				
35–40%	5 (4.4)	8 (7.3)				
< 35%	11 (9.7)	15 (13.8)				
EF change (%)	− 0.22 ± 4.3	0.4 ± 5.6	0.336	**–**	**–**	**–**
Diastolic dysfunction grade						
0	4 (3.7)	6 (5.7)	0.651^b^	**–**	**–**	**–**
1	101 (94.4)	98 (92.4)				
2	1 (0.9)	2 (1.9)				
3	1 (0.9)	0 (0.0)				
PAP	26.0 ± 6.9	25.1 ± 7.0	0.364	**–**	**–**	**–**
Total cost	237.8 ± 57.5	289.2 ± 88.3	** < 0.001**	22.7	− 0.2, 45.6	0.052
Insurance cost	191.5 ± 59.6	236.0 ± 71.1	** < 0.001**	18.8	− 1.8, 39.3	0.074
Patient cost	34.7 ± 33.8	35.3 ± 20.4	0.878	**–**	**–**	**–**

Bold values are significant

Data are presented as n (%) or mean ± standard deviation

*Coef.* linear regression coefficient; *CVA* cerebrovascular accident; *EF* ejection fraction; *GI* glycemic index; *IABP* intra-aortic balloon pump; *ICU* intensive care unit; *LOS* length of stay; *MI* myocadial infarction; *OR* odds ratio; *PAP* pulmonary artery pressure; *PCI* percutaneous coronary intervention

^a^Based on Kruskal–Wallis test

^b^Based of Fisher’s exact test

^c^Adjusted for ejection fraction values at before surgery using ANCOVA test

^d^Adjusted for body mass index, ejection fraction at before surgery, history of dyslipidemia, history of COPD, history of PCI, history of aspirin and statin consumption, presence of mitral regurgitation and application of primary PCI before surgery using multiple logistic or multiple linear regression tests

### Six month follow up

Findings from 6-month follow-up are presented in Table 3. Number of readmissions (p = 0.801), cases requiring angiography (p = 0.100), cases requiring re-PCI (p = 0.156), need for re-CABG (p > 0.999) and CVA (p > 0.999) did not differ between the two groups. During the 6-month follow-up period, seven out-of-hospital deaths were observed, 2 of which were in the aspirin group and 5 in the DAPT group, which did not differ significantly between the two groups (p = 0.446).

**Table 3:** Outcome of patients treated with DAPT or aspirin alone in follow up (after 6 months)

Parameter	Aspirin alone	DAPT	p
Haemoglobin (mg/dL)	10.3 ± 1.4	10.2 ± 1.3	0.930
Platelet (n/mm^3^)	284.7 ± 106.5	281.8 ± 101.8	0.831
Cr (mg/dL)	1.3 ± 1.0	1.2 ± 0.9	0.889
Ejection fraction in follow up (%)	45.8 ± 11.0	43.9 ± 12.2	0.608^a^
≥ 55%	7 (28.0)	8 (28.6)	0.436^b^
40–54%	15 (60.0)	12 (42.9)	
35–40%			
< 35%	3 (12.0)	8 (28.6)	
Ejection fraction change (%)	1.0 (6.0)	0 (9.6)	0.656
Re-admission			
No	107 (93.0)	105 (92.1)	0.787
Yes	9 (7.0)	9 (7.9)	
Number of need to re-angiography			
0	114 (99.1)	109 (95.6)	0.100^b^
1	0 (0.0)	4 (3.5)	
2	1 (0.9)	1 (0.9)	
Number of need to re-PCI			
0	113 (100.0)	111 (98.2)	0.498^b^
1	0 (0.0)	2 (1.8)	
Need to re-CABG	0 (0.0)	0 (0.0)	–
GI bleeding	0 (0.0)	1 (0.9)	> 0.999
CVA	0 (0.0)	1 (0.9)	> 0.999
Death in follow up	2 (1.7)	5 (4.4)	0.446^c^

*Cr* creatinine; *CVA* cerebrovascular accident; *GI* gastrointestinal; *PCI* percutaneous coronary intervention

^a^Adjusted for ejection fraction values at before surgery using ANCOVA test

^b^Based on Kruskal–Wallis test

^c^Based of Fisher’s exact test

## Discussion

Basis of treatment for MI is the use of DAPT with the aim of reducing coagulation and ischemic events [7, 23], on the other hand, this strategy increases the risk of bleeding [10, 11], thus there is disagreement between cardiologists and cardiovascular surgeons regarding DAPT before CABG. The aim of this prospective cohort study was to evaluate the effect of DAPT with aspirin and clopidogrel up to the day before CABG on postoperative outcomes in patients with MI. The findings of the present study showed that preoperative DAPT compared to mono-APT did not significantly affect any of the postoperative in-hospital outcomes (level of hemoglobin, mild or severe bleeding, blood and number of pack cell transfusion, cardio-bypass and cross-clamp time, number of death, CVA, duration and costs of hospitalization, EF level, diastolic dysfunction grade and PAP level). Also, after 6 months, none of the outcomes (number of cases requiring readmissions, angiography, re-PCI, re-CABG or number of CVA cases) were affected. In other words, DAPT has no effect on postoperative outcomes of CABG beyond mono-APT.

The pharmacological efficacy of clopidogrel varies from person to person due to the presence of a polymorphism in cytochrome P4502C19 (the major determinant of the coagulation response to clopidogrel inhibition of the P2Y12 receptor) [24, 25]. The unpredictability of the response to clopidogrel due to the presence of a polymorphism in cytochrome P4502C19, slow onset of efficacy and lasting inhibitory effect on the P2Y12 receptor, has led to cessation of use of this drug for less than 5 days in CABG candidate patients. This is a risk factor for bleeding during and after surgery [26]. Therefore, most studies are based on DAPT cessation (clopidogrel discontinuation) 5 days before CABG. Few studies, such as ours, were designed based on DAPT administration less than 5 days before CABG. Among these studies, one study examined only the DAPT group versus the group without antiplatelet therapy, and there was no comparison between dual and mono-antiplatelet therapy [27]. In other studies, the low sample size [26] and the lack of adequate outcomes (especially mortality, cardiac and cerebral events, length of stay in the ICU after surgery) [28] has caused not all aspects of this question to be covered. In 2015, Plicner et al., conducted a study on CABG candidate patients (under DAPT with aspirin and Plavix) in which study the DAPT continued until the day before CABG. The aim of their study was to investigate the relationship between preoperative platelet aggregation and bleeding during and after CABG. The results of their study showed that if light transmission aggregometry (LTA) in patients with DAPT was greater than or equal to 50%, the risk of bleeding, transfusions, and chest opening to control bleeding was comparable to that of patients receiving aspirin alone and discontinuation of Plavix 5 days before CABG does not appear to be necessary in all CABG candidate patients. The results of their study show that the main factor in predicting adverse outcomes following CABG is not mono or dual-antiplatelet therapy strategy, but preoperative platelet aggregation reasonably predicts outcomes during and after CABG. They reported LTA < 50% as predictors of bleeding, transfusions, and chest opening (to control bleeding) during and after CABG [26]. The results of our study are consistent with this study and no difference was observed between the postoperative outcomes of patients undergoing DAPT or aspirin alone. However, in our study, preoperative platelet aggregation was not measured for patients. Based on the comparison of our results with the results of the study of Plicner et al., it can be concluded that based on careful examination of coagulation function (by gold standards such as LTA) in patients, mono or dual-antiplatelet therapy is determined before CABG surgery and not solely based on DAPT effects. This could be one of the limitations of our study that patients’ coagulation function was not evaluated before DAPT administration. Another study by Mahla et al., in 2012 found similar results to the Plicner study. In this study DAPT was administered with aspirin and clopidogrel until the day before CABG. The aim of this study was to investigate the role of coagulation function in patients receiving DAPT and CABG candidates. The results of their study showed that the strategy of accurately measuring coagulation function by thrombelastography in patients receiving DAPT with aspirin and clopidogrel reduces the waiting time for CABG surgery without increasing the risk of intraoperative and postoperative bleeding. Their study confirms that attention to coagulation function status in CABG candidate patients is more important than adopting mono or dual-antiplatelet therapy before surgery. They concluded from their study that the rate of intraoperative and postoperative bleeding in patients receiving aspirin and clopidogrel was not different from that in patients receiving aspirin alone [28]. The results of our study also confirm the findings of the study of Mahla et al., although our study did not examine coagulation function. In another study conducted by Heymann et al., in 2005, DAPT was prescribed before CABG surgery up to 2 days before surgery. The results of their study showed that DAPT with aspirin and clopidogrel up to 2 days before CABG significantly increased drainage loss after CABG surgery. They also showed that if the DAPT was stopped earlier than 2 days before the operation, the drainage loss rate in the DAPT group was still higher, but this increase was not significant. It is noteworthy that in the study of Heymann et al., their study did not have an aspirin group alone and a significant increase in drainage loss after CABG surgery was reported compared to the group without antiplatelet therapy [27]. If their study defined an aspirin group, it would most likely yield results such as the study of Plicner et al., Mahla et al., as well as our study. Charif et al., conducted a retrospective study in 2019 to assess the safety of DAPT up to the time of CABG. In this study, similar to our study, patients in the control group received only aspirin and patients in the DAPT group received clopidogrel in addition to aspirin. Contrary to our study, in the DAPT group, in addition to anticoagulants, platelets were infused prophylactically. The results of this study on the safety of DAPT and the main outcomes were consistent with the results of our study so that there was no significant difference in the bleeding, reoperation and the number of in-hospital deaths in the DAPT group compared to the control group [29]. A major difference between the study of Charif et al., and ours was the intraoperative administration of platelets in addition to DAPT. If the study team considered a non-platelet-DAPT group in their study in addition to the control and DAPT groups with platelet administration, the results of their study would be better able to judge whether DAPT was safe before CABG surgery. Also, the results of our study were more comparable with the results of their study.

Among the limitations of the present study is the small sample size compared to similar studies in this field, which can be an important confounding factor in generalizing the results to the population of CABG candidate patients. Other confounding factors include the single-center and unblinded nature of this study. In the present study, matching of the studied groups was not performed, which could be the source of potential selection bias in the study. In addition, the number of patients studied was not the same for all variables (because of the lost to follow up). One of the limitations of this study is the follow-up period of patients after discharge from the hospital (6 months), which was done only by telephone. In this study, there are significant differences in baseline variables which must be addressed with propensity score matching to minimise bias and create a balanced dataset, allowing a simple and direct comparison of baseline covariates between treated and untreated patients. However, there is not a consensus on using propensity score instead of conventional regression method to overcome baseline differences among studied groups. According to clinical prediction references, empirical comparisons did not indicate the superiority of propensity score methods over conventional regression analysis for confounder adjustment. We adjusted all potential confounders in the multivariable logistic regression model and the current logistic regression model had acceptable goodness of fit measures. We decided not to use the propensity score matching method since we worried about “the propensity score paradox”. For example, imagine that in a dataset dependent variable is almost sample independent of two potential confounders. In this case, the propensity score will hardly vary with the value of the two potential confounders. Matching subjects with normal to abnormal values using the propensity score than amounts to essentially randomly picking a control. On average, this randomly picked control will have covariate values further away from the abnormal subject’s values, then in the original full sample or if you were to match based on a covariate distance metric like Euclidean distance. As such, it is argued that propensity score matching can increase confounder imbalance, thereby leading to estimates of exposure effects with greater bias. According to these reasons, we only used the conventional adjusting method for controlling the potential confounder [30, 31]. Duration and dose of DAPT is one of the important questions in this field that should be considered in designing future studies. Given these explanations, the elimination of confounders and limitations mentioned in this study seems necessary in the design of future studies so that the results can be more generalizable to the population of CABG candidate patients by clinicians and policymakers.

## Conclusion

A 6-month follow-up in this study showed that DAPT with aspirin and clopidogrel before CABG in patients with MI has no effect on postoperative outcomes more than mono-APT with aspirin. Given that DAPT plays a key role in preventing ischemic events in ACS patients, so based on the results of this study, the use of DAPT strategy in the management of ACS patients can be used with more confidence.

## Data Availability

The datasets used and/or analysed during the current study are available from the corresponding author on reasonable request.

## Abbreviations

ACS: Acute coronary syndrome
CABG: Coronary artery bypass grafting
CPB: Cardiopulmonary bypass
CSICU: Cardiac surgery intensive care unit
CVA: Cerebrovascular accident
DAPT: Dual antiplatelet therapy
EF: Ejection fraction
MI: Myocardial infarction
NSTEMI: Non-ST-segment elevation myocardial infarction
OR: Odds ratio
PCI: Percutaneous coronary intervention
STEMI: ST-segment elevation myocardial infarction
